# Comprehensive assessment of goat adipose tissue-derived mesenchymal stem cells cultured in different media

**DOI:** 10.1038/s41598-024-58465-1

**Published:** 2024-04-10

**Authors:** Michelle Abraham, Ibraz Kori, Utkarsha Vishwakarma, Sandeep Goel

**Affiliations:** 1 DBT-National Institute of Animal Biotechnology (NIAB), Hyderabad, Telangana India; 2 DBT-Regional Centre for Biotechnology (RCB), Faridabad, Haryana India

**Keywords:** Animal biotechnology, Stem-cell biotechnology

## Abstract

Mesenchymal stem cells (MSCs) have demonstrated potential in treating livestock diseases that are unresponsive to conventional therapies. MSCs derived from goats, a valuable model for studying orthopaedic disorders in humans, offer insights into bone formation and regeneration. Adipose tissue-derived MSCs (ADSCs) are easily accessible and have a high capacity for expansion. Although the choice of culture media significantly influences the biological properties of MSCs, the optimal media for goat ADSCs (gADSCs) remains unclear. This study aimed to assess the effects of four commonly used culture media on gADSCs’ culture characteristics, stem cell-specific immunophenotype, and differentiation. Results showed that MEM, DMEM/F12, and DMEM-LG were superior in maintaining cell morphology and culture parameters of gADSCs, such as cell adherence, metabolic activity, colony-forming potential, and population doubling. Conversely, DMEM-HG exhibited poor performance across all evaluated parameters. The gADSCs cultured in DMEM/F12 showed enhanced early proliferation and lower apoptosis. The cell surface marker distribution exhibited superior characteristics in gADSCs cultured in MEM and DMEM/F12. In contrast, the distribution was inferior in gADSCs cultured in DMEM-LG. DMEM/F12 and DMEM-LG culture media demonstrated a significantly higher potential for chondrogenic differentiation and DMEM-LG for osteogenic differentiation. In conclusion, DMEM/F12 is a suitable culture medium for propagating gADSCs as it effectively maintains cell morphology, growth parameters, proliferation and lower apoptosis while exhibiting desirable expression patterns of MSC-specific markers. These findings contribute to optimising culture conditions for gADSCs, enhancing their potential applications in disease treatment and regenerative medicine.

## Introduction

Mesenchymal stem cells (MSCs) have shown promise in treating various disease conditions in livestock unresponsive to conventional therapies. These cells play a significant role in tissue repair and regeneration through multiple mechanisms, including cellular differentiation to replace injured cells and release cytokines and growth factors. Although MSCs can be isolated from different sources, such as adipose tissue^1^, bone marrow^2^, umbilical cord^3^, placenta^4^, amniotic fluid^5^, and amniotic membrane^6^, they can be easily accessed and isolated in large quantities with minimally invasive harvesting procedures from adipose tissue due to its abundance in the body. Adipose tissue-derived mesenchymal stem cells (ADSCs) exhibit high expansion potential in vitro, making them useful in stem-cell-based therapies and tissue engineering^7–9^. ADSCs have been reported to treat musculoskeletal disorders which do not respond to conventional treatment^10^, such as osteoarthritis^11,12^, injuries of the tendo-ligamentous apparatus^13,14^, and bone fractures^15,16^ in companion and large animals.

There has been growing interest in MSCs derived from goats due to their potential applications in regenerative medicine, tissue engineering, and disease modelling^17,18^. Goats are valuable large animal models for human diseases and serve to bridge the gap between small animal studies and clinical trials^18^. Similarities with humans in organ size, physiology, and metabolism make goat-derived MSCs a clinically relevant model for studying diseases and assessing the effectiveness and safety of potential therapies^19^. Furthermore, goats are frequently employed as animal models in orthopaedic research because they resemble humans in bone structure and biomechanics^20^. Research using goat-derived MSCs can provide valuable insights into bone formation, fracture healing, and osteoarthritis mechanisms. Additionally, it can facilitate the development of innovative strategies for bone tissue engineering, skeletal regeneration^21,22^ and healing of fractures by promoting the secretion of paracrine factors^23^.

Since MSCs can be obtained from various sources, their nutritional requirements may vary. Several studies have suggested that the choice of culture media significantly influences the biological properties of MSCs^24^. In vitro culture of MSCs is performed using a defined basal culture medium (BCM). Optimal BCM and culture conditions support cell growth and proliferation and regulate cellular processes such as apoptosis and senescence^25^. Previous evidence suggests that the selection of BCM significantly impacts the biological characteristics of MSCs^24,26^. BCMs with diverse formulations are commercially available for culturing MSCs, and the selection of BCMs varies among laboratories. These BCMs predominantly comprise inorganic salts, glucose, amino acids, vitamins, and trace elements essential for cellular growth and survival. Notably, when bone marrow MSCs (BMSCs) were cultured in different BCMs such as modified Eagle's medium (MEM), Dulbecco’s MEM (DMEM), DMEM with 1000 mg/ml glucose (DMEM-LG), DMEM with 4500 mg/ml glucose (DMEM-HG), and Iscove’s modified Dulbecco’s medium (IMDM), it was observed that MEM yielded the highest cell count, with marginal differences in phenotype across subsequent passages^26^. Another study exploring BCMs found that a modification of the “Verfaillie” medium with DMEM-HG resulted in increased adherence of BMSC cell populations and higher proliferation rates of BMSCs in early passages, outperforming other media formulations, including MEM and DMEM-LG-based media^24^. In a separate investigation comparing four BCMs, ADSCs exhibited enhanced proliferation when MEM and DMEM-LG were employed, with no notable alterations in morphology and viability during subsequent passages^27^. However, the goat adipose-derived MSCs (gADSCs) have been cultivated in various basal media, including MEM^28–32^, DMEM-LG^20,33–42^, Dulbecco’s MEM/ Nutrient Mixture F12 (DMEM/F12)^43–52^ and DMEM-HG^53^, which media best supports gADSCs cell growth and proliferation remains elusive. The optimal selection of BCM can be contingent on the type and quantity of MSCs under cultivation. It is important to consider the impact of BCM on the proliferative capacity of MSCs and the preservation of their intrinsic characteristics while selecting the ideal BCM for their MSC cultures. Therefore, it is essential to identify a BCM suitable for the viability, proliferation, differentiation potential, and overall functionality of gADSCs.

Hence, we conducted this study to assess the effect of four frequently employed BCMs on various aspects of gADSCs, including cell morphology, culture characteristics, surface marker distribution, apoptosis, chondrogenic and osteogenic differentiation potential. Instead of solely examining specific culture additives, our investigation focused on analysing the overall compositions of routinely utilised media in our laboratory. Our objective was to understand the impact of these compositions on the cultural characteristics of gADSCs.

## Results

### Isolation and morphological evaluation of gADSCs

The isolated cells were quantified and assessed for viability using trypan blue dye exclusion. A total of 2.2 ± 0.2 × 10^6^ cells/g of adipose tissue were successfully isolated, with cell viability of 94.2 ± 2.1%. These isolated cells were then seeded at a concentration of 2 × 10^4^ cells per cm^2^ in four different culture media and passaged at least three times prior to evaluation.

**Figure 1:**
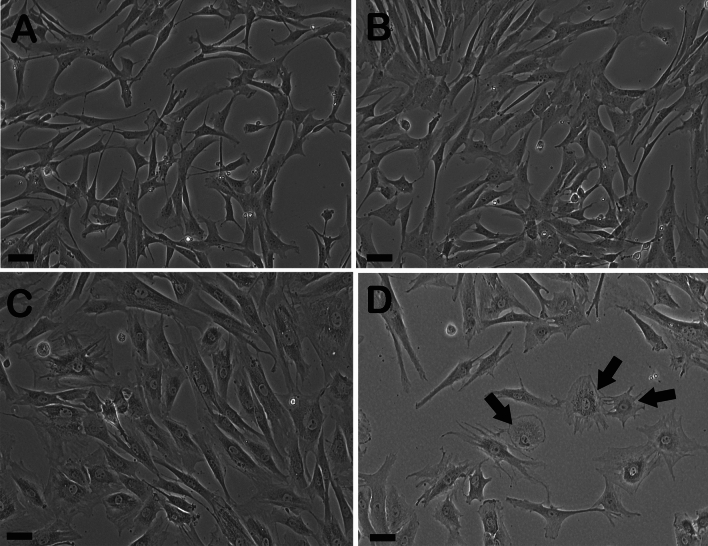
Morphological evaluation of gADMSCs cultured in four different BCMs with 10% FBS. Phase contrast image of gADMSCs cultured in (**A**) MEM, (**B**) D/F12, (**C**) D-LG and (**D**) D-HG. Arrows indicate cells cultured in D-HG flattened and misshaped with irregular contours, cytoplasmic vacuolations and extensions. Scale bar = 20 µm.

When seeded in the DMEM-high glucose (D-HG) medium, gADSCs exhibited reduced adhesion to the culture substrate, leading to cell detachment or floating. However, cells seeded in MEM, DMEM/F12 (D/F12), and DMEM-low glucose (D-LG) adhered firmly to the culture substrate. Morphological analysis of gADSC cultures in MEM, D/F12, and D-LG revealed a well-spread fibroblast-like morphology characterised by elongated or spindle-shaped cells. The gADSCs displayed a small, rounded nucleus with a clear nuclear membrane and thin cytoplasmic extensions. They were evenly distributed and maintained intercellular contact with neighbouring cells. The gADSCs demonstrated a homogeneous population with minimal cell shape and size variations with no apparent differences between the three culture media (Fig. 1A–C). However, gADSCs cultured in the D-HG medium could not be cultured beyond five passages and exhibited a lower cell count than expected and cells with flattened and misshapen morphology, irregular contours and cytoplasmic extensions (Fig. 1D). Furthermore, the nuclei showed signs of abnormality, including enlarged size and irregular shape, while the cytoplasm appeared vacuolated.

### Cell adherence, adherent cell viability, metabolic activity (MTT), colony formation assay (CFA), population doubling time (PDT), and intracellular reactive oxygen species (ROS) analysis of gADSCs

**Figure 2:**
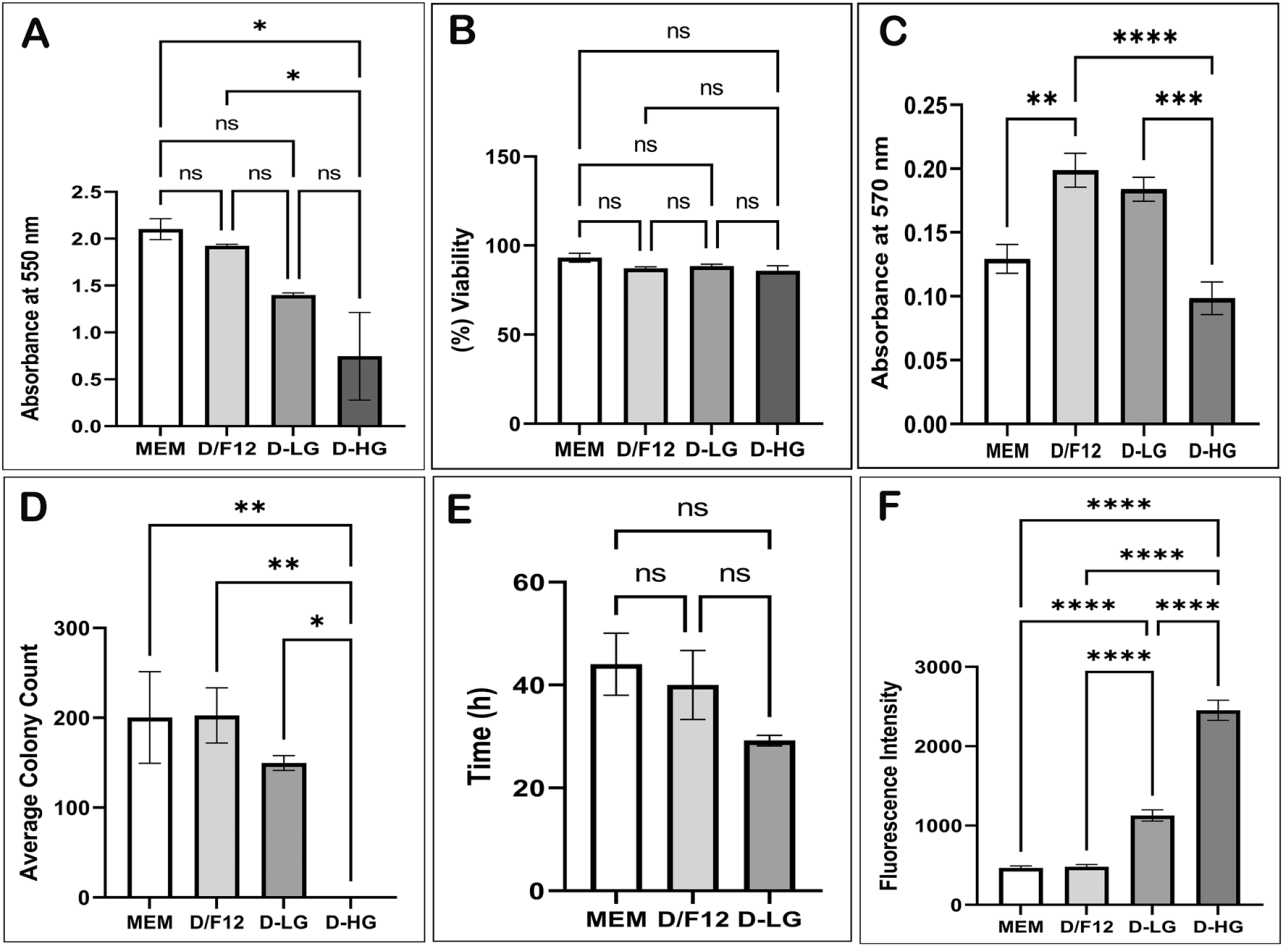
Culture characterisation of gADSCs cultured in different BCMs. (**A**) Cell adherence, (**B**) adherent cell viability, (**C**) MTT, (**D**) CFU, (**E**) PDT and (**F**) ROS assay. Data are presented as mean ± SEM (*P < 0.05, **P < 0.01, ***P < 0.001 and ****P < 0.0001).

Adherence of gADSCs to plastic surface exhibited variation when cultured in four distinct BCMs (Fig. 2A). Notably, gADSCs cultured in D-HG displayed significantly diminished adhesion compared to those cultured in MEM or D/F12 (P < 0.05). However, no significant decrease was observed in the adherence of gADSCs cultured in D-LG compared with those cultured in D-HG (P > 0.05). Similarly, no statistically significant difference was discerned in the adherence of gADSCs cultured in MEM, D/F12, and D-LG (P > 0.05).

The assessment of the viability of adherent gADSCs, cultivated in four distinct BCMs, was performed using calcein-AM staining (Fig. 2B). Remarkably, no statistically significant difference was observed in the percentage of viability among gADSCs cultured in any of the BCMs (P > 0.05). Similarly, the percentage of PI-positive cells was consistently below 0.4%, with no discernible differences among gADSCs cultured in the four BCMs (P > 0.05; data not presented).

The metabolic activity of gADSCs cultured in four different BCMs was assessed using the 3-(4,5-dimethylthiazol-2-yl)-2,5-diphenyltetrazolium bromide (MTT) assay (Fig. 2C). The results demonstrated a significant increase in metabolic activity in D/F12 and D-LG compared with that in MEM and D-HG (P < 0.001). However, no significant difference was observed between the metabolic activity of gADSCs in D/F12 and D-LG (P > 0.05).

The clonogenic capacity of gADSCs across the four BCMs was evaluated. The findings from the CFA (Fig. 2D) revealed that the number of gADSC colonies observed in MEM (200.3 ± 50.9) and D/F12 (202.7 ± 30.8) was higher compared with that in D-LG (149.7 ± 8.3). However, the difference did not reach statistical significance (P > 0.05). Notably, no ADSC colonies were detected in D-HG.

In order to determine the time required for gADSCs to double in number within a specific BCM, the PDT was measured (Fig. 2E). Remarkably, the PDT of ADSCs was found to be the shortest in D-LG (29.21 ± 1.03 h). However, no statistically significant difference was observed between the PDT of gADSCs in MEM (44.06 ± 6.02 h), D/F12 (40.03 ± 6.69 h) and D-LG (P > 0.05). Due to the absence of clonogenic potential of ADSCs in D-HG, it was impossible to determine the PDT in this medium.

The ROS release assay was conducted to quantify the levels of intracellular ROS as a measure of oxidative stress (Fig. 2F). The findings revealed that ROS levels in gADSCs were significantly higher in D-HG followed by D-LG compared with that in MEM and D/F12 (P < 0.001).

### Proliferation and apoptosis assay of gADSCs

**Figure 3:**
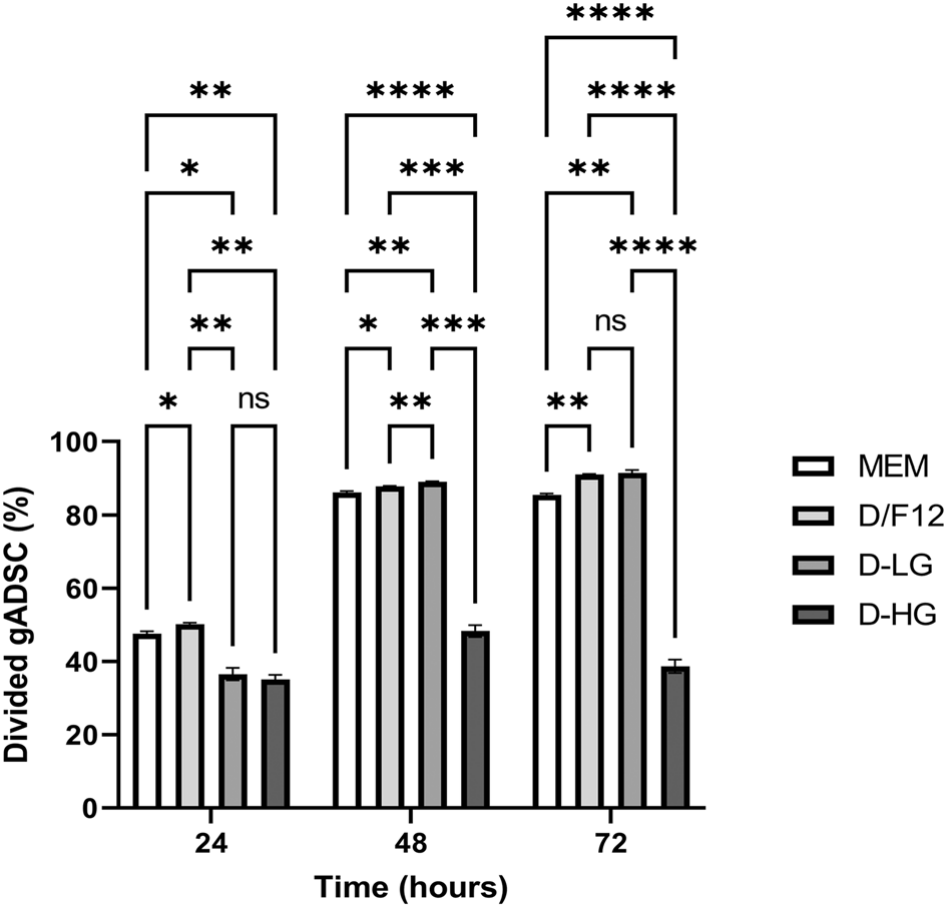
Assessment of gADSCs proliferation in various BCMs using the CFSE assay. Data are presented as mean ± SEM (*P < 0.05, **P < 0.01, ***P < 0.001 and ****P < 0.0001).

gADSC proliferation in four distinct BCMs was assessed by carboxyfluorescein diacetate succinimidyl ester (CFSE) staining. After 24 h post-seeding, the analysis revealed that the proportion of divided gADSCs was significantly lower in D-LG and D-HG compared to D/F12 and MEM (Fig. 3; P < 0.01). Likewise, the percentage of divided gADSCs in MEM was lower than that in D/F12 (P < 0.05). At 48-h post-seeding, the percentage of divided gADSCs was notably higher in D-LG than in all other BCMs (P < 0.001). However, 72-h post-seeding, the percentage of dividing gADSCs in D/F12 and D-LG was the highest and statistically similar (P > 0.05). Despite this, the percentage of dividing gADSCs in MEM remained lower than in D-LG and D/F12 (P < 0.01). Furthermore, the percentage of dividing gADSCs remained consistently lowest in D-HG at both 48- and 72-h post-seeding (P < 0.001).

**Figure 4:**
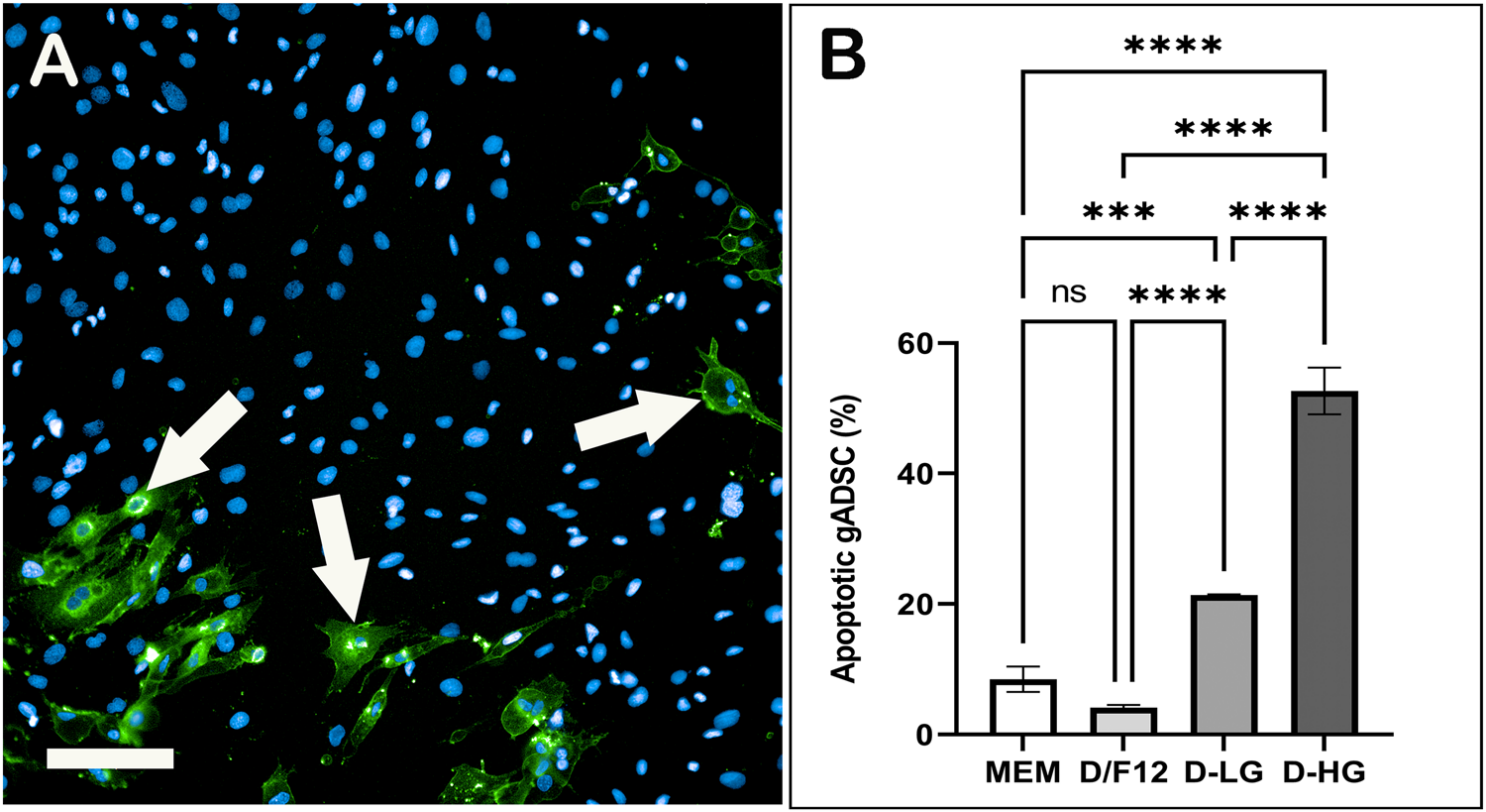
Estimating apoptotic gADSCs cultured in different BCMs by Annexin V/PI staining. (**A**) A representative image of gADSCs stained with Annexin V (green; arrows), PI (red, not present), and nuclei (blue). (**B**) Percentage of apoptotic gADSCs in different BCMs. Data are presented as mean ± SEM (*P < 0.05, **P < 0.01, ***P < 0.001 and ****P < 0.0001). Scale bar = 100 µm.

The gADSCs cultured in four BCMs were assessed for apoptosis using AnnexinV/PI staining (Fig. 4A). The results revealed that the percentage of apoptotic gADSCs was significantly higher in D-HG than in the other three BCMs (Fig. 4B, P < 0.0001). Furthermore, percentage of apoptotic gADSCs was also significantly higher in D-LG compared with that in MEM (P < 0.001) and D/F12 (P < 0.0001). The percentage of apoptotic gADSCs cultured in MEM and D/F12 was not different (P > 0.05). No significant difference was observed in the percentage of PI-positive gADSCs in four BCMs, and their percentage remained less than 0.2% (P > 0.05; data not shown).

Due to suboptimal performance in various culture parameters, gADSCs cultured in D-HG were not included in the MSC-specific surface markers analysis and bilineage differentiation assessment.

### MSC-specific surface markers analysis of the gADSCs

**Figure 5:**
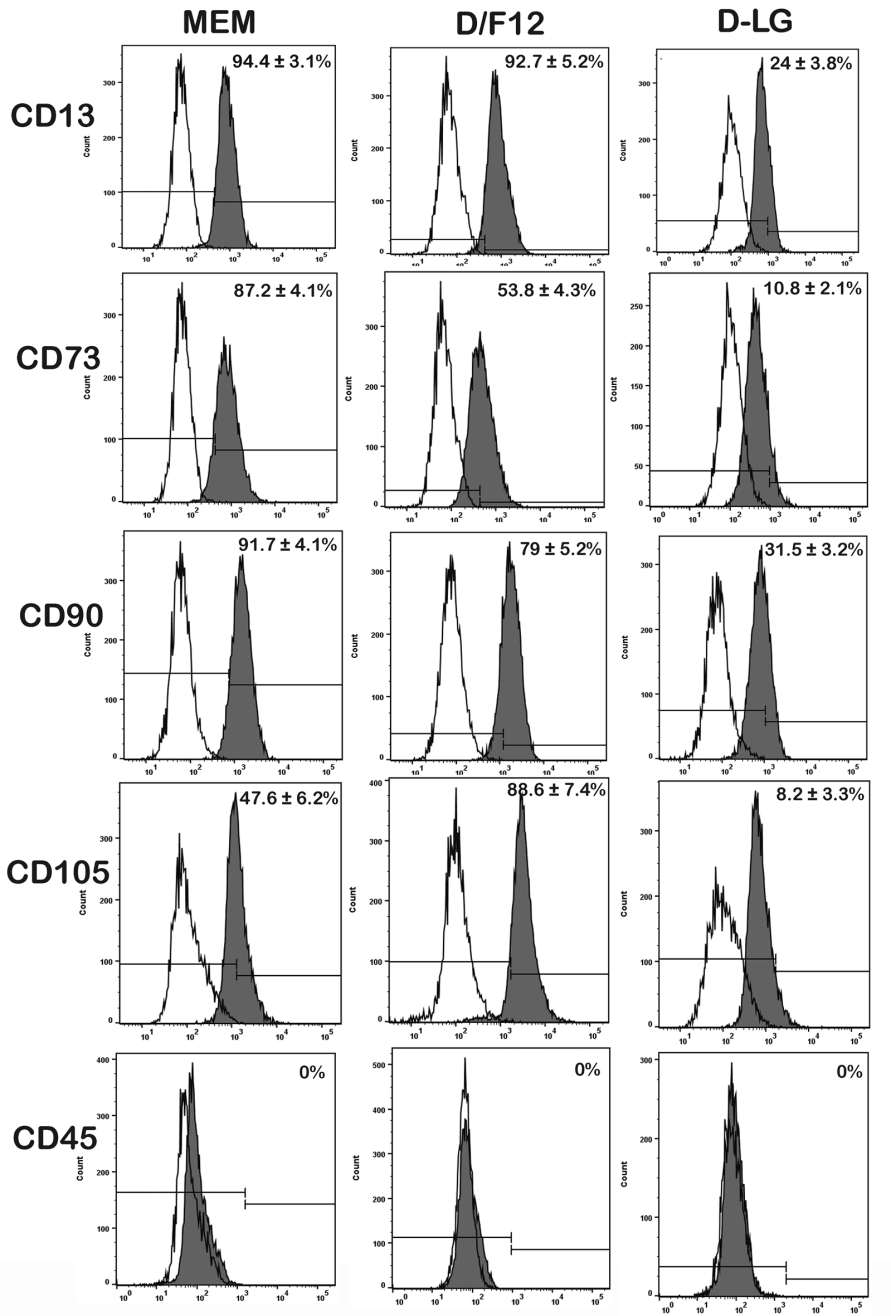
Flow cytometry analysis of CD13, CD73, CD90, CD105, and CD45 cell surface proteins on gADSCs cultured in different BCMs. Open histograms show unstained, and solid histograms show specific staining for the indicated marker. Three donor gADSCs were analysed at P4, each representative image for a given marker, and BCM incorporates the mean ± SEM value derived from three replicates.

Flow cytometry analysis was utilised for immunophenotyping MSC-specific surface markers on the gADSCs cultured in three different BCMs. The expression of MSC-specific cell surface markers and the number of cells expressing these markers varied (Fig. 5). The number of gADSCs expressing CD13, CD73, and CD90 was the highest in MEM, followed by D/F12 and the lowest in D-LG. However, the number of gADSCs expressing CD105 was the highest in D/F12, followed by MEM, and the lowest in D-LG. Importantly, CD45 expression in gADSCs was absent in all three media.

### Bilineage differentiation of gADSCs

**Figure 6:**
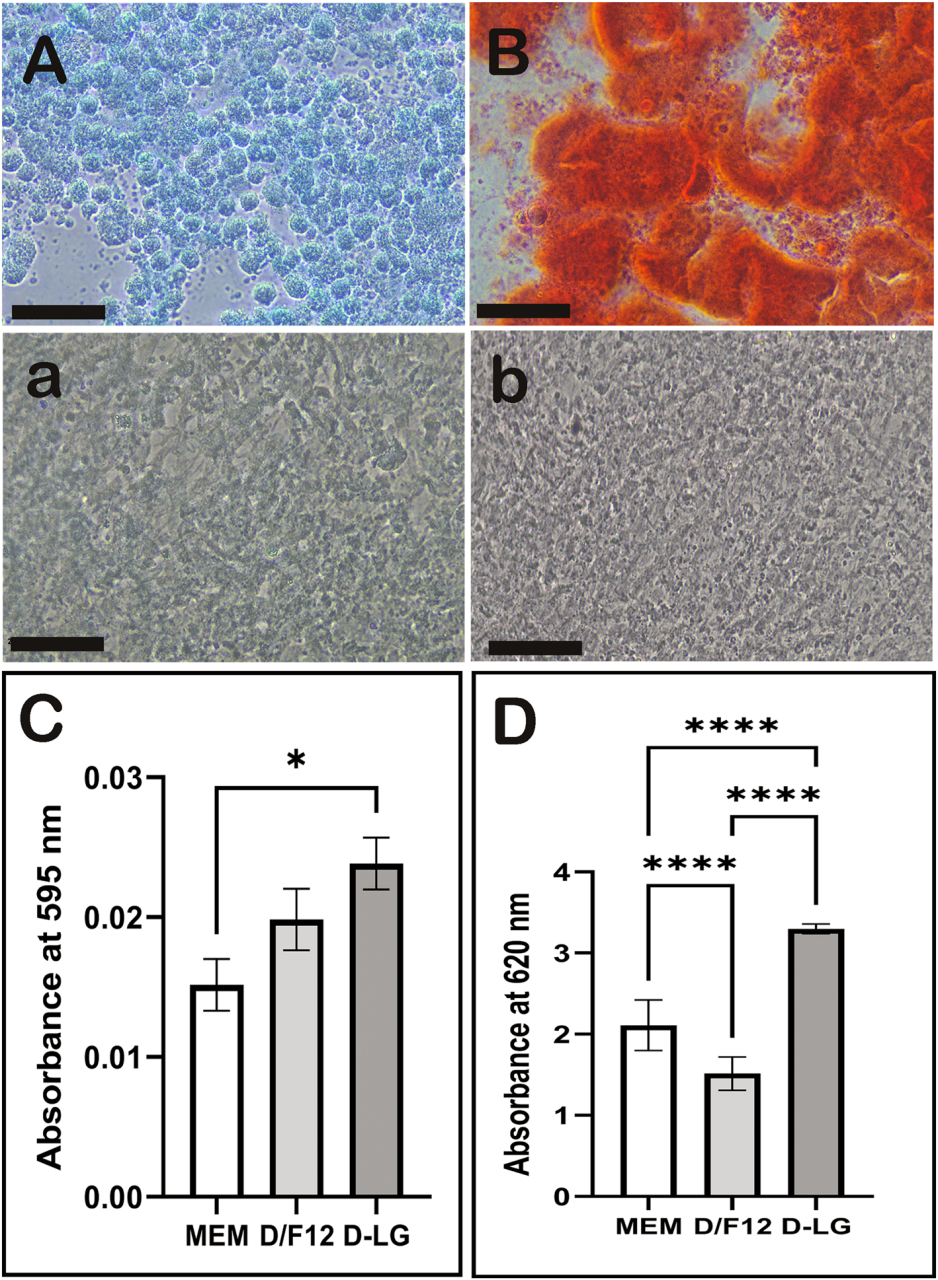
Bilineage differentiation of gADSCs cultured in different BCMs. Representative image of (**A**) chondrogenic differentiated gADSCs stained with alcian-blue and (**B**) osteogenic differentiated gADSCs stained with alizarin-red stains. (**a**) and (**b**) are gADSCs cultured in complete media and stained with alcian-blue and alizarin-red as a negative control. Dye absorption measurements for (**C**) alcian-blue and (**D**) alizarin-red in gADSCs at day 21 of chondrogenic and osteogenic induction culture. Data are presented as mean ± SEM (*P < 0.05 and ***P < 0.001).

The potential of gADSC to differentiate into chondrogenic and osteogenic lineages was evaluated in three different BCMs by alcian blue and alizarin red staining (Fig. 6A,B). gADSCs cultured in complete media, without the induction of differentiation, did not show chondrogenic and osteogenic lineages-specific staining (Fig. 6a,b). Dye absorption quantification was conducted to assess the potential for bilineage differentiation quantitatively. Compared with the other media, chondrogenic differentiation of gADSC was significantly lower in MEM (Fig. 6C, P < 0.05). No significant difference in the potential of gADSCs for chondrogenic differentiation was observed between D/F12 and D-LG (P > 0.05). Conversely, the quantification of alizarin dyes demonstrated that the potential of gADSC for osteogenic differentiation was higher in D-LG than in D/F12 (Fig. 6D, P < 0.001). Moreover, the potential of gADSCs for osteogenic differentiation was higher in MEM than in D/F12 (P < 0.01).

## Discussion

The study presented comprehensive insights into gADSCs cultured in different BCMs, including their morphological characteristics, adherence, viability, metabolic activity, clonogenic capacity, proliferation, apoptosis, surface marker expression, and differentiation potential. These findings have significant implications for optimising the culture conditions of gADSCs in applications such as regenerative medicine.

Morphological evaluation of gADSCs cultured in different BCMs revealed distinct cell adhesion and morphologic differences. Cells cultured in MEM, D/F12, and D-LG displayed well-spread fibroblast-like morphology with elongated or spindle-shaped cells. In contrast, gADSCs cultured in D-HG exhibited abnormal morphology and vacuolated cytoplasm. Furthermore, gADSCs cultured in D-HG demonstrated reduced adhesion, leading to cell detachment or floating, suggesting that this medium may not support their growth and maintenance. Similarly, human bone marrow-derived mesenchymal stem cells (BM-MSCs) cultured in D-HG have shown abnormal morphological changes and decreased proliferation capacity beyond a few passages^54^. In contrast, BM-MSCs cultured in a modified medium supplemented with D-HG have exhibited better adherence and initial proliferation rates than in other media, such as MEM and D-LG^24^. However, multiple reports have reported that shifting MSCs to D-HG negatively affects their cellular functions, reduces colony-forming activity, increases apoptosis, and causes premature senescence^55–57^.

Interestingly, despite the high glucose concentration in D/F12, gADSCs exhibited optimal morphology. This finding suggests that glucose concentration alone may not control MSC proliferation, and other components, including amino acids, vitamins, and inorganic salts, may play a crucial role in maintaining the multilineage differentiation potential and sustaining the high proliferation capacity of MSCs (Table 1). Further experiments are necessary to comprehensively understand the role of these components in the culture media and their impact on the cellular functions and characteristics of gADSCs.

**Table 1:** Comparison of composition among MEM, D/F12, D-LG, and D-HG (in mg/L).

Components	MEM	D/F12	D-LG	D-HG
Inorganic salts				
Calcium chloride (Anyd.)	200.00	116.60	200.00	200.00
Calcium chloride.2H_2_O	–	–	-	-
Cupric sulfate	–	0.0013	–	–
Ferric nitrate	–	0.05	0.10	0.10
Ferric sulfate	–	0.41	–	–
Magnesium sulfate	97.67	48.84	97.67	97.67
Magnesium chloride	–	28.64	–	–
Potassium chloride	400.00	311.80	400.00	400.00
Sodium chloride	6800.00	6995.50	6400.00	6400.00
Sodium bicarbonate	2200.00	2438.00	3700.00	3700.00
Sodium phosphate, monobasic	140.00	62.50	125.00	125.00
Sodium phosphate, dibasic	–	71.02	–	–
Zinc sulfate	–	0.43	–	–
Other compounds				
d-Glucose	1000.00	3151.00	1000.00	4500.00
Hypoxanthine	–	2.39	–	–
Linoleic acid	–	0.042	–	–
Lipoic acid	0.20	0.105	–	–
Phenol red	10.00	8.10	15.00	15.00
Putrescine-2HCl	–	0.081	–	–
Sodium pyruvate	110.00	55.00	110.00	–
Thymidine	–	0.365	–	–
Amino acids				
l-Alanine	25.00	4.45	–	–
l-Arginine-HCl	126.98	147.50	84.00	84.00
l-Arginine	﻿–	–	–	–
l-Asparagine-H_2_O	50.00	7.50	–	–
l-Aspartic acid	30.00	6.65	–	–
l-Cysteine/L-Cysteine 2HCl	-	31.29	63.00	63.00
l-Cysteine-HCl-H_2_O	100.00	17.56	–	–
l-Glutamic acid	75.00	7.35	–	–
l-Glutamine	292.00	365.00	584.00	584.00
Glycine	50.00	18.75	30.00	30.00
l-Histidine	﻿–	–	–	–
l-Histidine HCl-H_2_O	42.00	31.48	42.00	42.00
l-Isoleucine	52.40	54.47	105.00	105.00
l-Leucine	52.00	59.05	105.00	105.00
l-Lysine	﻿–	–	–	–
l-Lysine-HCl	73.00	91.25	146.00	146.00
l-Methionine	15.00	17.24	30.00	30.00
l-Phenylalanine	32.00	35.48	66.00	66.00
l-Proline	40.00	17.25	–	–
l-Serine	25.00	26.25	42.00	42.00
l-Threonine	48.00	53.45	95.00	95.00
l-Tryptophan	10.00	9.02	16.00	16.00
l-Tyrosine disodium salt/disodium salt dihydrate	52.00	55.79	104.00	104.00
l-Valine	46.00	52.85	94.00	94.00
Vitamins				
l-Ascorbic acid	50.00	–	–	–
Biotin	0.10	0.0035	–	–
d-Calcium pantothenate	1.00	2.24	4.00	4.00
Choline chloride	1.00	8.98	4.00	4.00
Folic acid	1.00	2.65	4.00	4.00
i-Inositol	2.00	12.60	7.20	7.20
Niacinamide	1.00	2.02	4.00	4.00
Pyridoxal.HCl/Pyridoxine HCl	1.00	2.01	4.00	4.00
Riboflavin	0.10	0.219	0.40	0.40
Thiamine-HCl	1.00	2.17	4.00	4.00
Vitamin B12	1.36	0.68	–	–

MSCs adhere strongly to plastic surfaces in culture, facilitating their isolation and expansion in vitro. This property is essential for their in vitro manipulation and expansion in research and clinical applications. Adhesion is critical in cell growth, differentiation, and survival and is a necessary component of cell differentiation. The adherence ability and adherent viability of gADSCs cultured in different BCMs were assessed, and the results revealed that although the adherent cells were significantly lower in gADSCs cultured in D-HG than in other BCMs, the viability of gADSCs was not low. The lower cell adherence of gADSCs in D-HG can be attributed to the high glucose and composition of D-HG^24^. Additional research is imperative to comprehensively understand the specific components within the BCM and their consequential influence on the adherence and viability of gADSCs.

The metabolic activity of gADSCs was assessed using the MTT assay, which measures cell viability and metabolic function by evaluating the activity of mitochondrial enzymes within the MSCs. The results revealed that D/F12 and D-LG media supported a considerably higher metabolic activity than MEM and D-HG. These findings contradict previous reports that indicated no significant variation in the proliferation or viability of MSCs in low or high-glucose media^58–61^. Interestingly, a study investigating different culture media demonstrated that an adapted form of the “Verfaillie” medium supplemented with D-HG exhibited a higher proliferation rate of BM-MSC in the initial passages compared with that in other MEM and D-LG media^24^. However, in our study, gADSCs cultured in MEM, which has a similar glucose concentration to D-LG, exhibited a lower metabolic activity. These findings suggest that D/F12 and D-LG may provide a more favourable microenvironment for gADSCs for their metabolic activity, regardless of the glucose concentrations used. This inconsistency could be due to other components in the media, which may have influenced the metabolic activity of gADSCs. It could also be attributed to the difference in the origin of the stem cells or the animal species used. Comprehensive experiments are crucial for understanding the significance of these factors and their impact on the metabolic activity of gADSCs.

The clonogenic capacity of gADSCs, referring to their ability to form colonies from single cells, was evaluated using the CFA. While no significant differences were observed, gADSCs cultured in MEM and D/F12 exhibited more colonies than those cultured in D-LG. These results are consistent with those from an earlier report, indicating that human ADSCs cultured in D-LG and D/F12 produced significantly more colonies than those cultured in D-HG^62^. Similarly, human BM-MSCs cultured in MEM showed the highest cell yield, with minor differences in their phenotype over subsequent passages^26^. In another study comparing different basal media, human ADSCs displayed increased proliferation in MEM and D-LG without noticeable changes in their morphology and viability during subsequent passages^27^. Notably, no colonies were formed when gADSCs were cultured in D-HG, indicating its unsuitability for supporting the clonogenicity of gADSCs. Recent reports support our findings, highlighting that media containing high glucose concentration diminish the clonogenicity of MSCs compared with those with lower glucose concentration^54,57^. These results collectively suggest that MEM and D/F12 are more conducive for clonogenicity of gADSCs, while D-LG supports moderate colony formation. On the other hand, D-HG negatively impacts the clonogenicity of gADSCs.

We determined growth curves and population doubling time, which allowed for assessing cell proliferation functions in the three different culture media. However, no significant differences (P > 0.05) were observed among the media. Due to its inability to support colony formation, clonogenicity of gADSCs could not be determined in D-HG. These findings are consistent with previous reports, indicating that D-HG is unsuitable for promoting the growth and proliferation of MSCs^54,57^. However, it is noteworthy that a previous study did not observe any differences in colony-forming assays or population doubling time of porcine BM-MSC cultured in MEM, D-LG, and D-HG^63^. This inconsistency could be attributed to variations in cell origin and animal species used in the current study.

The level of oxidative stress in gADSCs was evaluated by measuring the intracellular ROS levels using a ROS assay. The results indicated that gADSCs cultured in D-HG and D-LG exhibited significantly higher ROS levels than cells cultured in MEM and D/F12. While high glucose levels have been linked to increased ROS production in MSCs^64^, the elevated ROS level observed in gADSCs cultured in D-LG was unexpected, which could potentially be credited to the absence of specific antioxidants such as ascorbic acid and lipoic acid in D-LG (Table 1). These antioxidants have been shown to delay senescence in MSCs by regulating ROS and AKT/mTOR signalling pathways^65^. Additionally, alpha-lipoic acid, a potent antioxidant, has been found to reduce ROS levels and inhibit apoptosis in mature human pluripotent stem cell-derived hematopoietic stem/progenitor cells^66^. Amino acids such as l-proline, l-glutamic acid, and l-cysteine were present in MEM and D/F12 but absent from D-LG and D-HG. These precursors may have enhanced the antioxidant properties of MEM and D/F12^67–69^. It is noteworthy that D-LG also contains added iron (III) nitrate (Fe[NO_3_]_3_), which can generate highly reactive ‘free’ iron ions that possess strong pro-oxidant properties, similar to other transition metal ions^70^. Further experiments and investigations are needed to thoroughly examine the role of these additional factors and their impact on controlling ROS levels in gADSCs.

CFSE is an effective and widely employed method for monitoring the division of lymphocytes^71,72^. Additionally, it is a valuable tool for assessing the proliferation of MSCs^73–75^. CFSE covalently attaches to enduring intracellular molecules, imparting them with the fluorescent dye carboxyfluorescein. Consequently, as a CFSE-labeled cell undergoes division, its progeny inherit half the CFSE-tagged molecules. This assay allows the measurement of each cell division by observing the corresponding reduction in cell fluorescence using flow cytometry. Our findings indicate that 24-h post-seeding, MEM and D/F12 effectively support the proliferation of gADSCs. However, at 48-h post-seeding, gADSCs exhibit the highest proliferation in D-LG, and by 72-h post-seeding, D-LG and D/F12 demonstrate similar percentages of gADSC proliferation. Intriguingly, gADSCs consistently exhibit poor proliferation in D-HG at all analysed time points. The poor proliferation in D-HG can again be attributed to the high glucose content, significantly reducing cell proliferation^55,56^. It can also be attributed to the absence of sodium pyruvate in D-HG, which is known for maintaining cell proliferation^76^. An elevated ROS level could also be a reason for poor proliferation in D-HG. However, additional investigations are warranted to substantiate these assertions. In summary, D/F12 emerges as the most suitable basal cell medium for supporting the early and late-stage proliferation of gADSCs.

Apoptosis in gADSCs cultured in various media was assessed using Annexin V labelling and high-content live-cell imaging^77^, revealing distinct staining patterns dependent on the BCMs. During apoptosis, the early loss of plasma membrane asymmetry exposes phosphatidylserine (PS) residues on the outer leaflet, a common occurrence across cell types^78^. Annexin V, with a specific affinity for PS, facilitates apoptosis detection by targeting the loss of plasma membrane asymmetry^79^. Our investigation demonstrated that gADSCs in D/F12 displayed the lowest percentage of Annexin V-positive cells, while D-HG cultures exhibited the highest Annexin V staining. Notably, D-LG-cultured gADSCs showed a significantly greater Annexin V staining cell count than those in D/F12 and MEM. The increased occurrence of apoptotic cells in D-HG and D-LG environments may be attributed to the absence of critical factors, such as lipoic acid^80^, vitamin B12^81,82^ and biotin^83,84^, which are recognised for their role in mitigating apoptosis. Additionally, elevated ROS levels in D-LG and D-HG may contribute to heightened apoptosis in gADSCs, as increased ROS is implicated in MSC apoptosis^85^. Nonetheless, these hypotheses require further experimental validation for conclusive confirmation.

FACS analysis was employed to evaluate the expression of MSC-specific cell surface markers in gADSCs cultured in various media. However, gADSCs cultured in D-HG exhibited suboptimal performance in multiple culture parameters, leading to their exclusion from the evaluation of MSC-surface markers and bilineage differentiation. The present study unveiled notable variations in CD13, CD73, CD90, and CD105 expression levels among gADSCs cultured in different media. Similarly, when diverse media were compared in the context of BM-MSCs culture, significant alterations were observed in the expression profile of MSC-specific cell surface markers^24,86^. The present study demonstrated variations in the number of cells expressing MSC-specific markers depending on the culture media used. Specifically, MEM supported the highest expression of CD13, CD73, and CD90, while D/F12 exhibited the highest expression of CD105.

In contrast, gADSCs cultured in D-LG showed poor expression of all markers compared to those cultured in MEM or D/F12. This reduced expression of cell surface markers in D-LG may be attributed to the higher levels of ROS present in the medium, as elevated ROS levels have been associated with decreased expression of MSC-specific markers^87^. However, a previous report indicated no difference in the expression of surface markers in human MSCs cultured in either MEM, D-LG or D-HG^62^. Our results are also inconsistent with those from another study in which the expression of MSC-specific markers after multiple passages was not impacted by the basal media^27^. Similarly, porcine BM-MSCs cultured in MEM and D-LG showed no difference in the expression of surface markers^63^. The inconsistent results in our study may be attributed to specific culture requirements of gADSCs, as evidenced by variations in the expression of cell surface markers. Importantly, none of the four culture media induced the expression of the hematopoietic marker CD45 in gADSCs, which is consistent with previous reports^87,88^.

The potential of gADSCs to differentiate into osteogenic and chondrogenic lineages was assessed using three distinct culture media. The findings revealed that the choice of culture medium significantly impacted the differentiation outcomes. Chondrogenic differentiation was notably lower in MEM than in D/F12 and D-LG. Similarly, a previous study reported a considerably diminished potential of BM-MSCs for chondrogenic differentiation in MEM compared with that in D-LG and D/F12^24^. Conversely, osteogenic differentiation was significantly higher in D-LG than in the other media, while it was higher in MEM than in D/F12. A prior investigation concluded that D-LG served as the most suitable medium for both expansion and differentiation of the osteogenic lineage, resulting in a robust osteogenic response^88^. An increased potential of gADSCs for osteogenic differentiation in D-LG could be attributed to elevated ROS levels. A previous study demonstrated that tonsil-derived MSCs, persistently exposed to mild ROS levels through the ROS-hydrogel system, promoted bone regeneration^89^. However, the reason for a lower osteogenic differentiation of gADSCs cultured in D/F12 remains unclear. It is plausible that specific additives unique to D/F12 may have rendered gADSCs resistant to osteogenic differentiation. For instance, recent research has indicated that vitamin B12-deficient media stimulated osteogenesis in human MSCs^90^. Vitamin B12 is an essential component of D/F12 but is absent in D-LG. Thus, the presence of vitamin B12 in D/F12 may have hindered the osteogenic differentiation of gADSCs. Nevertheless, a comprehensive evaluation of other cultural media components may provide further insights into their specific roles in differentiating gADSCs.

Our goal was to compare four commonly used BCMs across different laboratories, emphasising a broader perspective rather than isolating the effects of individual components. This approach enhances our understanding of how seemingly routine choices influence experimental outcomes. Our findings reveal that DMEM/F12 supports both early and late gADSC proliferation, maintaining cell morphology and growth parameters with lower apoptosis. Furthermore, DMEM-LG was found suitable for differentiating gADSCs into osteogenic lineages. Indeed, DMEM (high glucose and low glucose) and DMEM/F12 exhibit distinct characteristics. While the precise molecular mechanisms underlying the influence of each medium on the property of gADSC remains incompletely understood, it is evident that the choice of culture medium can impact various aspects, including cell adhesion, morphology, metabolic activity, clonogenic capacity, proliferation, surface marker expression, and differentiation potential of gADSCs. These findings contribute to optimising culture conditions for gADSCs in diverse applications, such as regenerative medicine and tissue engineering. Future studies should focus on isolating and investigating individual media components to attribute observed differences accurately.

## Materials and methods

Unless otherwise stated, all chemicals and reagents used in the present study were obtained from Sigma–Aldrich Chemical Company (St. Louis, MO, USA).

### Isolation of gADSCs

Adipose tissues (AT) were collected immediately from the inguinal region from freshly slaughtered goats (N = 4) from a local slaughterhouse and transferred to phosphate buffer saline (PBS) containing 1× antibiotic–antimycotic (AA; 100 U penicillin, 0.1 mg streptomycin and 0.25 μg Amphotericin B/ml) and transported on ice to the laboratory. After arriving at the laboratory, the AT was rinsed with PBS and kept in PBS containing AA with mild shaking at 4 °C to eliminate contamination. After 1 h, the AT was transferred to the bio-safety cabinet and washed thrice with PBS. Approximately ten grams of AT was placed in a sterile Petri dish and minced thoroughly into small pieces (~1 mm^3^) using a sterile scissor. Minced tissue was transferred into a 50 ml centrifuge tube and resuspended in a digestion mixture containing 1 mg/ml collagenase type II (Gibco) in PBS. The digestion mixture was incubated at 37 °C in the shaking water bath (100 rpm) for 30 min. After the tissue digestion, the digestion mixture was filtered through a 100 µm cell strainer (Corning). The filtrate was centrifuged at 726×*g* for 5 min, resulting in a stromal vascular fraction (SVF) pellet. The supernatant was discarded, and the SVF was resuspended in 5 ml PBS supplemented with 10% fetal bovine serum (FBS; Gibco) and AA. The cell pellet was spin-washed thrice with PBS and resuspended in an erythrocyte lysis buffer for 2 min. The reaction was halted by the addition of twice the volume of PBS with 10% FBS, and the cell was counted using a hemocytometer, with cell viability assessed using the trypan blue dye exclusion method and was seeded at a 2 × 10^4^ cells/cm^2^ density in four different BCMs, i.e., Minimum Essential Medium (MEM), Dulbecco’s Modified Eagle Medium/Nutrient Mixture F12 (DMEM/F12), Dulbecco’s Modified Eagle Medium with low glucose (D-LG) and Dulbecco's Modified Eagle Medium with high glucose (D-HG; all from Gibco) with 10% FBS (Gibco) and 1× AA (complete media) at 37 °C in a humid and 5% CO_2_ environment.

### Culture of gADSCs

Once the confluency of seeded cells reached approximately 80–90%, cells were trypsinised and subcultured in the same media. Briefly, the confluent cells were washed twice with PBS and treated with a working trypsin–EDTA solution (0.05% trypsin and 0.02% EDTA) for 2–5 min in a humidified environment at 37 °C, and trypsin activity was halted with a complete medium (medium containing 10% FBS with 1× AA). The recovered cells were centrifuged at 726×*g* for 5 min and subcultured. The medium was changed every 48 h until the cells reached 80–90% confluence. The cells were cultured and passaged in each culture medium thrice before analysis. All analyses were carried out in P4-6.

### Assessment of cell adhesion and adherent cell viability of gADSCs

A cell adhesion assay was performed for gADSCs cultured in four BCMs as described previously^91^. A total of 1 × 10^4^ cells/well were seeded in four different culture media in 96-well plates. Plates were incubated for 24 h and stained with 0.5% (w/v) crystal violet solution in methanol. The staining process consisted of aspirating the growth media, washing with PBS, fixing with 10% neutral formalin solution for 10 min, washing with PBS, incubating with crystal violet solution for 10 min, and washing twice with distilled water. The OD at 550 nm was measured by a microplate reader (Perkin Elmer Enspire), and all the experiments were performed in triplicate.

The viability of cultured adherent gADSCs was assessed using the calcein-acetoxymethyl ester (calcein-AM) and propidium iodide (PI) double staining (both from Thermo Fisher Scientific) as described previously^92^. Briefly, 1 × 10^4^ cells/well were seeded in four different culture media in a 96-well, black-walled, optically clear flat-bottom tissue-culture treated dish (PhenoPlate, PerkinElmer). Plates were incubated for 24 h, rinsed twice with PBS and incubated with calcein-AM (2 μM) and PI (1 μM) in PBS for 30 min at 37 °C. After incubation, the cells were rinsed with PBS and counterstained with Hoechst 33342 for 10 min for nuclear visualisation. The high-content analysis system Operetta CLS (PerkinElmer) captured and analysed the images using Harmony 5.1 software (PerkinElmer).

### Assessment of cell metabolic activity of gADSCs

The cell metabolic activity of gADSCs cultured in different BCMs was measured using an MTT assay. Briefly, cells were seeded into a 96-well culture plate (TPP; 1 × 10^4^ cells/well) and cultured for 48 h. Next, 10 μl MTT solution was added to each well. After 4 h incubation at 37 °C, 150 μl formazan solvent (4 mM HCl, 0.1% NP40 in isopropanol) was added into each well, and the culture plate was placed in a rocking bed for 10 min at a low-speed oscillation. The optical density (OD) at 570 nm was measured by a microplate reader (Perkin Elmer Enspire), and all the experiments were performed in triplicate.

### Colony formation assay (CFA) of gADSCs

For CFA, gADSCs expanded in different BCMs to 70–90% confluency and were harvested with trypsin–EDTA and counted using a hemocytometer. Colony-forming efficiency was evaluated by plating gADSCs at a clonogenic level of 1000 ± 10 cells in a 10 cm cell culture dish with 10 ml culture media. Samples were cultured undisturbed for 14 days and then stained with crystal violet to detect cell colonies (≥ 50 cells).

### Assessment of gADSCs population doubling time (PDT)

The PDT of gADSCs was evaluated to determine the proliferation rate cultured in different BCMs. The cell doubling time of gADSCs at P3 were calculated every 24 h for 192 h. Briefly, cell viability was assessed by the trypan blue exclusion test (> 98 ± 4%), and 3.5 × 10^4^ cells/well were seeded in a 6-well cell culture plate in the given medium. After every 24 h, the cells were detached, and cell viability was assessed (> 98 ± 3.1%) and counted using a hemocytometer. The PDT of the cells was calculated according to the formula, PDT = t × [ln2/(ln Nt − ln N0)] where t is the time for cell culture (unit: h), Nt is the number of cells after the culture (t), and N0 is the number of cells initially inoculated^93^.

### Measurement of the gADSCs intracellular reactive oxygen species (ROS) level

According to the manufacturer's instructions, the intracellular ROS level was measured in gADSCs cultured in different BCMs using 2,7-dichlorodihydrofluorescein diacetate (H2DCFDA; Invitrogen). After reaching 80% confluency, the cells were washed with PBS and incubated with PBS containing 10 μM H2DCFDA at 37 °C for 30 min. The fluorescence intensity was analysed using a microplate reader (Perkin Elmer Enspire), excitation and emission  at 495 nm and 525 nm wavelengths, respectively. All the experiments were performed in triplicate.

### Assessment of gADSCs proliferation

The effects of different BCMs on the proliferation of gADSC were evaluated using CFSE labelling assay as described previously^73^. Briefly, 4 × 10^6^ gADSCs cultured in respective culture media were harvested and stained with 2.5 µM CFSE (CellTrace™ CFSE Cell Proliferation Kit, Invitrogen). CFSE labelling was terminated by adding complete culture media containing 10% FBS. Cells were washed with complete culture media twice and analysed after 10 min of incubation for 0-h reading or seeded in T25 culture flasks at 2 × 10^4^ cells/cm^2^ density for analysis at 24, 48, and 72 h. Cells were harvested at each indicated time point, and data was acquired by flow cytometry (FACSMelody; BD Biosciences) and data analyses were performed using the FlowJo 10.8.1 software. The peak obtained on 0-h reading was gated as the undivided population, and the percentage of divided cells at each time point was obtained.

### Assessment of gADSCs apoptosis

The percentage of cultured apoptotic gADSCs in four different BCMs was assessed utilising Annexin V/PI staining. gADSCs were seeded at a density of 1 × 10^4^ cells/well in a 96-well, black-walled, optically clear flat-bottom tissue-culture treated dish (PhenoPlate, PerkinElmer). After 48 h, the culture medium was aspirated, and cells were washed with PBS. Subsequently, cells were stained using the Dead Cell Apoptosis Kit with Annexin V Alexa Fluor 488 and PI (Thermofisher Scientific) according to the manufacturer's guidelines. In brief, cells were rinsed with ice-cold PBS and then incubated in 200 μl of binding buffer. Ten microliters of Annexin V stock solution were added to the cells and incubated for 15 min at room temperature. The cells were then further incubated with 2 μl propidium iodide (PI) and counterstained with Hoechst 33342 for 10 min for nuclear visualisation. The high-content analysis system Operetta CLS (PerkinElmer) captured and analysed the images using Harmony 5.1 software (PerkinElmer).

### Flow cytometry analysis

Flow cytometry assessed the gADSC’s immune profile cultured in different culture media. 1 × 10^6^ gADSCs were fixed in ice-cold ethanol for 30 min on ice, washed thrice with PBS and suspended in a blocking buffer (0.5% BSA with 2% FBS in PBS). The gADSCs were incubated with the following fluorophore-conjugated primary antibodies as per the manufacturer’s protocol; CD45 (PE, Invitrogen, MA1-81458), CD13 (FITC, Invitrogen MA1-35080), CD73 (FITC, Bio Legend, BL-344015), CD90 (FITC, SAB4700706-100TST) and CD105 (FITC, Bio Legend, BL-323203). Cells stained with isotype control IgG conjugated to FITC or PE were negative controls. The labelled cells were washed thrice with FACS buffer (0.5% BSA and 0.05% Sodium Azide in PBS) and resuspended in 500 µL FACS buffer analysed on BD FACSMelody using Flowjo 10.8.1 software.

### Bilineage differentiation of ADSCs and their quantification

As described earlier, the gADSCs cultured in three different BCMs until 60–80% confluency were dissociated using the trypsin–EDTA solution. The cell number was evaluated using a hemocytometer, and cell viability was assessed using trypan blue dye exclusion. The cells were seeded in a 12-well plate at 1 × 10^4^ cells/cm^2^ and incubated for 24 h at 37 °C in a humidified atmosphere of 5% CO_2_. gADSCs were induced with specific chondrogenic and osteogenic differentiation media (StemPro^®^, Gibco). The respective differentiation media replaced the cell culture media after cells had attained 60–70% confluence. Cells in a respective culture medium were a negative control for lineage differentiation. The media was changed every fourth day. The cells were cultured for 21 days, fixed in 4% PFA, and then stained using alcian-blue and alizarin-red stains for chondrogenic and osteogenic lineage confirmation, respectively.

The differentiation to chondrogenic and osteogenic lineages was determined by specific staining. Briefly, for chondrogenic staining, the cells were stained with 1% alcian-blue staining solution (Millipore, USA) overnight at 37 °C. The following day, the dye was washed off with deionised water and cell pictures were taken using an inverted microscope fitted with a camera (Nikon, Japan). The staining dye was extracted for the quantitative analysis of alcian-blue staining by incubating the stained cells with 6 M guanidine hydrochloride overnight at room temperature, and the absorbance was read at an OD of 595 nm. The samples with no cells and negative control cells were also stained with 0.05% alcian-blue staining solution, as described above, and staining dye was extracted and quantified for normalisation of reading. The cells were stained with 1% alizarin-red solution (pH 4.2) for 45 min at room temperature for osteogenic staining. The cells were washed with deionised water until the water did not appear orange. Calcified nodules were stained as red spots and were photographed by a microscope. For quantification of alizarin-red dye, the cells were dried at 37 °C, and the stain was solubilised within 10% cetylpyridinium chloride in 10 mM sodium phosphate (pH 7.0) by shaking for 15 min, and the absorbance was read at 620 nm. The calcium deposition is expressed as OD. The samples with no cells and negative control cells were also stained with 1% alizarin-red solution, as described above, and staining dye was extracted and quantified for normalisation of reading.

### Statistical analyses

The results were presented as mean ± SEM. The statistical analyses were performed by GraphPad Prism (version 9.5.1) using the ANOVA test. Normality and homogeneity of variances were checked and confirmed to match with parametric assumptions prior to running ANOVA. Significant differences between the means were determined by analysing the data using the Tukey honest significance difference (Tukey HSD) test. The level of significance was set at P < 0.05.

## Data Availability

All data generated or analysed during this study are included in this published article (and its Supplementary Information files).

## References

[1] Romanov YA, Darevskaya A, Merzlikina N, Buravkova L (2005). Mesenchymal stem cells from human bone marrow and adipose tissue: Isolation, characterization, and differentiation potentialities. Bull. Exp. Biol. Med..

[2] Fortier L, Nixon A, Williams J, Cable CS (1998). Isolation and chondrocytic differentiation of equine bone marrow-derived mesenchymal stem cells. Am. J. Vet. Res..

[3] Lee OK (2004). Isolation of multipotent mesenchymal stem cells from umbilical cord blood. Blood.

[4] In’t Anker PS (2004). Isolation of mesenchymal stem cells of fetal or maternal origin from human placenta. Stem Cells.

[5] Tsai MS, Lee JL, Chang YJ, Hwang SM (2004). Isolation of human multipotent mesenchymal stem cells from second-trimester amniotic fluid using a novel two-stage culture protocol. Hum. Reprod..

[6] Mihu CM, Rus Ciuca D, Soritau O, Susman S, Mihu D (2009). Isolation and characterization of mesenchymal stem cells from the amniotic membrane. Rom. J. Morphol. Embryol..

[7] Lee SC, Jeong HJ, Lee SK, Kim S-J (2015). Lipopolysaccharide preconditioning of adipose-derived stem cells improves liver-regenerating activity of the secretome. Stem Cell Res. Ther..

[8] Abumaree M, Al Jumah M, Pace RA, Kalionis B (2012). Immunosuppressive properties of mesenchymal stem cells. Stem Cell Rev. Rep..

[9] Wang M, Yuan Q, Xie L (2018). Mesenchymal stem cell-based immunomodulation: Properties and clinical application. Stem Cells Int..

[10] Przadka P (2021). The role of mesenchymal stem cells (MSCs) in veterinary medicine and their use in musculoskeletal disorders. Biomolecules.

[11] Yun S, Ku SK, Kwon YS (2016). Adipose-derived mesenchymal stem cells and platelet-rich plasma synergistically ameliorate the surgical-induced osteoarthritis in Beagle dogs. J. Orthop. Surg. Res..

[12] Shah K (2018). Outcome of allogeneic adult stem cell therapy in dogs suffering from osteoarthritis and other joint defects. Stem Cells Int..

[13] Carvalho Ade M (2013). Equine tendonitis therapy using mesenchymal stem cells and platelet concentrates: A randomized controlled trial. Stem Cell Res. Ther..

[14] Canapp SO (2016). The use of adipose-derived progenitor cells and platelet-rich plasma combination for the treatment of supraspinatus tendinopathy in 55 dogs: A retrospective study. Front. Vet. Sci..

[15] Franco GG, Minto BW, Dreibi RM, Costa Junior JS, Dias L (2021). Percutaneous application of allogeneic adipose-derived mesenchymal stem cell in dogs submitted to minimally invasive plate osteosynthesis of the tibia. Acta Cir. Bras..

[16] Herrera D, Lodoso-Torrecilla I, Ginebra MP, Rappe K, Franch J (2023). Osteogenic differentiation of adipose-derived canine mesenchymal stem cells seeded in porous calcium-phosphate scaffolds. Front. Vet. Sci..

[17] Dias IE (2022). Mesenchymal stem cell studies in the goat model for biomedical research—A review of the scientific literature. Biology.

[18] Gugjoo MB, Gugjoo MB, Pal A (2020). Mesenchymal Stem Cell in Veterinary Sciences.

[19] Harding J, Roberts RM, Mirochnitchenko O (2013). Large animal models for stem cell therapy. Stem Cell Res. Ther..

[20] Zhang C (2021). Combined hydrogel and mesenchymal stem cell therapy for moderate-severity disc degeneration in goats. Tissue Eng. Part A.

[21] Dias IR, Viegas CA, Carvalho PP (2018). Large animal models for osteochondral regeneration. Adv. Exp. Med. Biol..

[22] Hotham WE, Henson FMD (2020). The use of large animals to facilitate the process of MSC going from laboratory to patient-'bench to bedside'. Cell Biol. Toxicol..

[23] Pankajakshan D, Agrawal DK (2014). Mesenchymal stem cell paracrine factors in vascular repair and regeneration. J. Biomed. Technol. Res..

[24] Hagmann S (2013). Different culture media affect growth characteristics, surface marker distribution and chondrogenic differentiation of human bone marrow-derived mesenchymal stromal cells. BMC Musculoskelet. Disord..

[25] Nikolits I, Nebel S, Egger D, Kress S, Kasper C (2021). Towards physiologic culture approaches to improve standard cultivation of mesenchymal stem cells. Cells.

[26] Sotiropoulou PA, Perez SA, Salagianni M, Baxevanis CN, Papamichail M (2006). Characterization of the optimal culture conditions for clinical scale production of human mesenchymal stem cells. Stem Cells.

[27] Dhanasekaran M, Indumathi S, Rashmi M, Rajkumar JS, Sudarsanam D (2012). Unravelling the retention of proliferation and differentiation potency in extensive culture of human subcutaneous fat-derived mesenchymal stem cells in different media. Cell Prolif..

[28] Vertenten G (2009). Evaluation of an injectable, photopolymerizable, and three-dimensional scaffold based on methacrylate-endcapped poly(d, l-lactide-co-epsilon-caprolactone) combined with autologous mesenchymal stem cells in a goat tibial unicortical defect model. Tissue Eng. Part A.

[29] Lippens E (2010). Evaluation of bone regeneration with an injectable, in situ polymerizable Pluronic F127 hydrogel derivative combined with autologous mesenchymal stem cells in a goat tibia defect model. Tissue Eng. Part A.

[30] Schop D (2009). Growth, metabolism, and growth inhibitors of mesenchymal stem cells. Tissue Eng. Part A.

[31] Sousa RP (2021). In vitro activation and development of goat preantral follicles enclosed in ovarian tissue co-cultured with mesenchymal stem cells. Reprod. Sci..

[32] Arrivabene Neves C (2020). Culture of goat preantral follicles in situ associated with mesenchymal stem cell from bone marrow. Zygote.

[33] Rothenberg AR, Ouyang L, Elisseeff JH (2011). Mesenchymal stem cell stimulation of tissue growth depends on differentiation state. Stem Cells Dev..

[34] Petrella F (2014). Stem cell transplantation effectively occludes bronchopleural fistula in an animal model. Ann. Thorac. Surg..

[35] Zhang Y (2012). Isolation, characterization, and gene modification of dairy goat mesenchymal stem cells from bone marrow. In Vitro Cell. Dev. Biol. Anim..

[36] Yin H (2016). Induction of mesenchymal stem cell chondrogenic differentiation and functional cartilage microtissue formation for in vivo cartilage regeneration by cartilage extracellular matrix-derived particles. Acta Biomater..

[37] Mosca JD (2000). Mesenchymal stem cells as vehicles for gene delivery. Clin. Orthop. Relat. Res..

[38] Pratheesh MD (2017). Comparative study on characterization and wound healing potential of goat (*Capra*
*hircus*) mesenchymal stem cells derived from fetal origin amniotic fluid and adult bone marrow. Res. Vet. Sci..

[39] Li JW (2011). In vitro chondrogenesis of the goat bone marrow mesenchymal stem cells directed by chondrocytes in monolayer and 3-dimensional indirect co-culture system. Chin. Med. J..

[40] Pratheesh MD (2013). Isolation, culture and characterization of caprine mesenchymal stem cells derived from amniotic fluid. Res. Vet. Sci..

[41] Liu X (2010). Repairing goat tibia segmental bone defect using scaffold cultured with mesenchymal stem cells. J. Biomed. Mater. Res. B Appl. Biomater..

[42] Burdzinska A (2018). Intraurethral co-transplantation of bone marrow mesenchymal stem cells and muscle-derived cells improves the urethral closure. Stem Cell Res. Ther..

[43] Elkhenany H, Amelse L, Caldwell M, Abdelwahed R, Dhar M (2016). Impact of the source and serial passaging of goat mesenchymal stem cells on osteogenic differentiation potential: Implications for bone tissue engineering. J. Anim. Sci. Biotechnol..

[44] Dubey A (2022). Deducing insulin-producing cells from goat adipose tissue-derived mesenchymal stem cells. Cell. Reprogramm..

[45] Costa CRM (2019). Adipose stem cells in reparative goat mastitis mammary gland. PLoS One.

[46] Joseph A (2020). Mesenchymal stem cell-conditioned media: A novel alternative of stem cell therapy for quality wound healing. J. Cell. Physiol..

[47] Wei X (2019). Mesenchymal stem cell-loaded porous tantalum integrated with biomimetic 3D collagen-based scaffold to repair large osteochondral defects in goats. Stem Cell Res. Ther..

[48] Wang X (2017). Epigenetic modification differences between fetal fibroblast cells and mesenchymal stem cells of the Arbas Cashmere goat. Res. Vet. Sci..

[49] Ren Y (2014). Potential of adipose-derived mesenchymal stem cells and skeletal muscle-derived satellite cells for somatic cell nuclear transfer mediated transgenesis in Arbas Cashmere goats. PLoS One.

[50] Qiu P (2012). A dose-dependent function of follicular fluid on the proliferation and differentiation of umbilical cord mesenchymal stem cells (MSCs) of goat. Histochem. Cell Biol..

[51] Jena D (2020). Growth and proliferation of caprine bone marrow mesenchymal stem cells on different culture media. Tissue Cell.

[52] Zhou F (2018). Repair mechanism of mesenchymal stem cells derived from nasal mucosa in orbital fracture. Am. J. Transl. Res..

[53] Somal A (2021). Comparative analysis of the immunomodulatory potential of caprine fetal adnexa derived mesenchymal stem cells. Mol. Biol. Rep..

[54] Pal R, Hanwate M, Jan M, Totey S (2009). Phenotypic and functional comparison of optimum culture conditions for upscaling of bone marrow-derived mesenchymal stem cells. J. Tissue Eng. Regen. Med..

[55] Li YM (2007). Effects of high glucose on mesenchymal stem cell proliferation and differentiation. Biochem. Biophys. Res. Commun..

[56] Stolzing A, Coleman N, Scutt A (2006). Glucose-induced replicative senescence in mesenchymal stem cells. Rejuvenation Res..

[57] Liu Y (2020). The effect of high glucose on the biological characteristics of nucleus pulposus-derived mesenchymal stem cells. Cell Biochem. Funct..

[58] Kichenbrand C, Grossin L, Menu P, Moby V (2020). Behaviour of human dental pulp stem cell in high glucose condition: Impact on proliferation and osteogenic differentiation. Arch. Oral Biol..

[59] Weil BR, Abarbanell AM, Herrmann JL, Wang Y, Meldrum DR (2009). High glucose concentration in cell culture medium does not acutely affect human mesenchymal stem cell growth factor production or proliferation. Am. J. Physiol. Regul. Integr. Comp. Physiol..

[60] Aswamenakul K (2020). Proteomic study of in vitro osteogenic differentiation of mesenchymal stem cells in high glucose condition. Mol. Biol. Rep..

[61] Hankamolsiri W (2016). The effects of high glucose on adipogenic and osteogenic differentiation of gestational tissue-derived MSCs. Stem Cells Int..

[62] Ahearne M, Lysaght J, Lynch AP (2014). Combined influence of basal media and fibroblast growth factor on the expansion and differentiation capabilities of adipose-derived stem cells. Cell Regen..

[63] Antebi B (2019). Bench-to-bedside optimization of mesenchymal stem cell isolation, processing, and expansion for in vivo administration. Regen. Med..

[64] Oh JY (2018). High glucose-induced reactive oxygen species stimulates human mesenchymal stem cell migration through snail and EZH2-dependent E-cadherin repression. Cell. Physiol. Biochem..

[65] Yang M (2018). Ascorbic acid inhibits senescence in mesenchymal stem cells through ROS and AKT/mTOR signaling. Cytotechnology.

[66] Dong Y (2020). Alpha lipoic acid promotes development of hematopoietic progenitors derived from human embryonic stem cells by antagonizing ROS signals. J. Leukoc. Biol..

[67] Liang X, Zhang L, Natarajan SK, Becker DF (2013). Proline mechanisms of stress survival. Antioxid. Redox Signal..

[68] Kaul S, Sharma SS, Mehta IK (2008). Free radical scavenging potential of l-proline: Evidence from in vitro assays. Amino Acids.

[69] Lu SC (2013). Glutathione synthesis. Biochim. Biophys. Acta.

[70] Gutteridge JM (1985). The behaviour of caeruloplasmin in stored human extracellular fluids in relation to ferroxidase II activity, lipid peroxidation and phenanthroline-detectable copper. Biochem. J..

[71] Lyons AB, Parish CR (1994). Determination of lymphocyte division by flow cytometry. J. Immunol. Methods.

[72] Quah BJ, Warren HS, Parish CR (2007). Monitoring lymphocyte proliferation in vitro and in vivo with the intracellular fluorescent dye carboxyfluorescein diacetate succinimidyl ester. Nat. Protoc..

[73] Urbani S, Caporale R, Lombardini L, Bosi A, Saccardi R (2006). Use of CFDA-SE for evaluating the in vitro proliferation pattern of human mesenchymal stem cells. Cytotherapy.

[74] Kumar S, Vaidya M (2016). Hypoxia inhibits mesenchymal stem cell proliferation through HIF1alpha-dependent regulation of P27. Mol. Cell. Biochem..

[75] Lee JY (2022). Comparative analysis of mesenchymal stem cells cultivated in serum free media. Sci. Rep..

[76] Diers AR, Broniowska KA, Chang CF, Hogg N (2012). Pyruvate fuels mitochondrial respiration and proliferation of breast cancer cells: Effect of monocarboxylate transporter inhibition. Biochem. J..

[77] Gelles JD, Chipuk JE (2016). Robust high-throughput kinetic analysis of apoptosis with real-time high-content live-cell imaging. Cell Death Dis..

[78] Reutelingsperger CP, van Heerde WL (1997). Annexin V, the regulator of phosphatidylserine-catalyzed inflammation and coagulation during apoptosis. Cell. Mol. Life Sci..

[79] van Engeland M, Nieland LJ, Ramaekers FC, Schutte B, Reutelingsperger CP (1998). Annexin V-affinity assay: A review on an apoptosis detection system based on phosphatidylserine exposure. Cytometry.

[80] Byun CH (2005). Alpha-lipoic acid inhibits TNF-alpha-induced apoptosis in human bone marrow stromal cells. J. Bone Miner. Res..

[81] Karabulut D (2022). Vitamin B12 suppresses GADD153, prevents apoptosis and regulates the testicular function in methotrexate treated rat testis. Biotech. Histochem..

[82] Li EY (2019). Vitamin B1 and B12 mitigates neuron apoptosis in cerebral palsy by augmenting BDNF expression through MALAT1/miR-1 axis. Cell Cycle.

[83] Pan M (2022). Biotin alleviates hepatic and intestinal inflammation and apoptosis induced by high dietary carbohydrate in juvenile turbot (*Scophthalmus*
*maximus* L.). Fish Shellfish Immunol..

[84] Valenciano AI, Mayordomo R, de La Rosa EJ, Hallbook F (2002). Biotin decreases retinal apoptosis and induces eye malformations in the early chick embryo. Neuroreport.

[85] Rodrigues M, Turner O, Stolz D, Griffith LG, Wells A (2012). Production of reactive oxygen species by multipotent stromal cells/mesenchymal stem cells upon exposure to fas ligand. Cell Transplant..

[86] Haack-Sorensen M (2008). Comparison of different culture conditions for human mesenchymal stromal cells for clinical stem cell therapy. Scand. J. Clin. Lab. Investig..

[87] Hou J (2013). Autophagy prevents irradiation injury and maintains stemness through decreasing ROS generation in mesenchymal stem cells. Cell Death Dis..

[88] Grossner T, Haberkorn U, Hofmann J, Gotterbarm T (2022). Effects of different basal cell culture media upon the osteogenic response of hMSCs evaluated by (99m)Tc-HDP labeling. Int. J. Mol. Sci..

[89] Choi DH (2021). Tonsil-derived mesenchymal stem cells incorporated in reactive oxygen species-releasing hydrogel promote bone formation by increasing the translocation of cell surface GRP78. Biomaterials.

[90] Vaes BL (2009). Vitamin B(12) deficiency stimulates osteoclastogenesis via increased homocysteine and methylmalonic acid. Calcif. Tissue Int..

[91] Bahsoun S, Coopman K, Akam EC (2020). Quantitative assessment of the impact of cryopreservation on human bone marrow-derived mesenchymal stem cells: Up to 24 h post-thaw and beyond. Stem Cell Res. Ther..

[92] Favi PM (2013). Cell proliferation, viability, and in vitro differentiation of equine mesenchymal stem cells seeded on bacterial cellulose hydrogel scaffolds. Mater. Sci. Eng. C Mater. Biol. Appl..

[93] Ofner D (1992). Relationship between quantity of silver stained nucleolar organizer regions associated proteins (Ag-NORs) and population doubling time in ten breast cancer cell lines. Pathol. Res. Pract..

